# Robust Beamforming Design for Energy Efficiency and Spectral Efficiency Tradeoff in Multi-STAR-RIS-Aided C-HRSMA

**DOI:** 10.3390/s25226917

**Published:** 2025-11-12

**Authors:** Shiming Teng, Xinwei Lin, Yafeng Wang

**Affiliations:** 1 The Key Laboratory of Universal Wireless Communications, Ministry of Education, Beijing University of Posts and Telecommunications, Beijing 100876, China; shimingteng@bupt.edu.cn; 2 The Future Research Laboratory, China Mobile Research Institute, Beijing 100053, China; linxinwei@chinamobile.com

**Keywords:** RSMA, STAR-RIS, spectrum efficiency (SE), energy efficiency (EE), symbiotic radio

## Abstract

This paper investigates a simultaneous transmitting and reflecting reconfigurable intelligent surface (STAR-RIS)-assisted cognitive hierarchical rate-splitting multiple access (C-HRSMA) system to enhance the system performance under imperfect channel state information (ICSI). By exploiting the natural user grouping afforded by the STAR-RIS and its distinct channel manipulation capabilities for the transmission and reflection users, we effectively mitigate inter-group common stream interference within C-HRSMA, consequently facilitating the achievement of higher spectral efficiency. Subsequently, the design is formulated as a non-convex optimization problem that incorporates the phase-shift matrix of STAR-RIS, the beamforming vector of the base station, and the common rate allocation vector. To address this non-convex problem, an alternating optimization (AO) technique is employed to decouple the primary problem and solve the subproblems using S-procedure and successive convex approximation (SCA). The simulation results validate that the proposed algorithm exhibits superior SE and EE performance against benchmark algorithms.

## 1. Introduction

Symbiotic radio (SR) and rate-splitting multiple access (RSMA) technologies are prominent candidate solutions for addressing spectral-and-energy efficiency wireless applications in 6G. SR leverages the advantages of cognitive radio and ambient backscatter communication by employing backscatter devices to utilize licensed spectrum and the message-splitting capability of RSMA enables flexible management of inter-user interference [1,2]. Their effective integration can create new opportunities for Internet of Things (IoT) applications to achieve enhanced spectral efficiency and higher energy efficiency. By integrating RSMA with SR technology, passive devices with controlled phase shifts are utilized to achieve improved signal quality and throughput.

### 1.1. Related Work

Joint beamforming is a key mechanism to maximize system performance of simultaneous transmitting and reflecting reconfigurable intelligent surface (STAR-RIS)-aided RSMA system. In ref. [3], authors elucidate an alternating optimization framework using Lagrange multipliers to optimize active beamforming at the base station (BS), block length, and passive beamforming at the reconfigurable intelligent surface (RIS) in RSMA systems, aiming to maximize the achievable rate. Owing to the potential of the integration of SR and RSMA, many significant contributions have been made to the RIS-aided SR system with RSMA. The authors in [4] focus on maximizing the energy efficiency in a SR system with mobile edge computing by jointly optimizing the transmission power, the beamforming vector at the receiver and the local computing frequency. Ref. [5] specifically examines the advantages of RSMA over non-orthogonal multiple access (NOMA) and orthogonal multiple access (OMA) in terms of outage probability, block error rate (BLER), and achievable sum rate in a hybrid aerial full-duplex relaying system where the RIS is mounted on an unmanned aerial vehicle (UAV) relay. Additionally, ref. [6] provides a comprehensive investigation into the outage performance of a RSMA system in a RIS-assisted downlink. The study in [7] incorporated ultra-reliable low latency communications (URLLC) and RIS-aided RSMA to achieve optimal system energy efficiency under finite block length and packet error rate constraints. Refs. [8,9] focus on secure communication in RSMA systems assisted by STAR-RIS and provide design schemes for maximizing the covert communication rate.

The integration of STAR-RIS with SR technologies has exhibited significant potential in recent literature, primarily attributed to their synergistic contribution to wireless channel enhancement. The study in [10] addresses the total power consumption minimization problem in an active STAR-RIS-assisted symbiotic radio system subject to hardware impairments (HIs), proposing an alternating optimization (AO) framework that jointly designs the BS transmit beamformer and a STAR-RIS coefficients to achieve near-optimal performance. The work in [11] proposes an energy efficiency (EE) maximization framework for a STAR-RIS-enabled cell-free symbiotic radio system by jointly optimizing the active beamforming at the access points (APs) and the passive transmission/reflection beamforming at the STAR-RIS using an efficient AO technique. The article in [12] demonstrates a significant quality of service (QoS) improvement in a multi-user cellular-symbiotic radio network by leveraging an active-STAR-RIS and massive MIMO capabilities at the BS, utilizing NOMA to effectively overcome the inherent challenge of transmitting information from passive symbiotic backscatter devices (SBDs). The authors in [13] formulate and solve a transmit power minimization problem for a STAR-RIS-empowered symbiotic radio system, where a customized block coordinate descent algorithm is employed to jointly optimize the active beamforming vector and the STAR-RIS coefficients to meet users’ minimum rate requirements under practical phase correlation constraints. Beyond its conventional applications, SR has been actively integrated with emerging paradigms, such as Integrated Sensing and Communication (ISAC) [14,15] and satellite communication systems [16,17], thereby significantly extending its functional capabilities.

### 1.2. Contribution

Motivated by the effective power allocation capabilities of RSMA and the flexible radio propagation characteristics of SR, we consider the beamforming design for DL-RSMA framework with multiple backscatter devices (BDs), where the BS serves multiple DL users via a STAR-RIS aiming to achieve high spectral-and-energy efficiency performance. The introduced channel uncertainties caused by imperfect channel state information (ICSI) and considered phase-shift constraints render the optimal beamforming design problem intractable. The major contributions of this work are summarized as follows:

- We propose a tradeoff scheme between EE and SE in a C-HRSMA system that incorporates common rate allocation, BS beamforming, and RIS beamforming. This approach exploits the spatial clustering characteristic of user distribution to enhance EE and SE.
- Given the uncertainties from ICSI and the non-convex fractional structure of the SE-EE maximization problem, we propose an iterative-based robust joint beamforming algorithm with the S-procedure, the successive convex approximation (SCA) method and Dinkelbach’s approach.
- The simulation results validate that the integrated C-HRSMA algorithm exhibits superior performance in terms of both SE and EE.

The symbols adopted throughout this work are given in Table 1. Note that unbold scripts denote sets, unbold symbols denote scalar variables, bold lowercase symbols denote vector variables, and bold uppercase symbols denote matrix variables.

## 2. System Model

**Figure 1:**
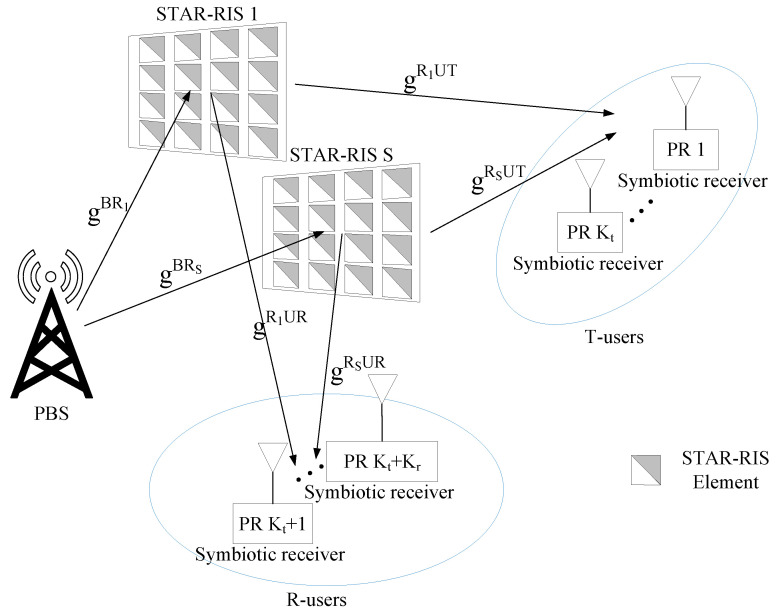
A multi-STAR-RIS-aided C-HRSMA system.

Consider a STAR-RIS-aided SR system where an *M*-antenna BS serves *K* single-antenna users via *S* STAR-RISs, each consisting of $N_{s}$ passive elements as shown in Figure 1. There are $K_{r}$ reflection users (R-users) and $K_{t}$ transmission users (T-users) in the reflection and transmission space of STAR-RIS, respectively. Overall, the STAR-RIS serves a set of $K=K_{t}+K_{r}$ users. Denoting $\Theta_{t}=diag\left(\theta_{t}\right)$ and $\Theta_{r}=diag\left(\theta_{r}\right)$ as the transmission and reflection coefficient matrices for the STAR-RIS operating under energy-splitting mode, respectively, such that $\theta_{t}=\left[\begin{array}{c} \nu_{t_{1}}\exp\left(j\phi_{t_{1}}\right),\ldots ,\nu_{t_{N}}\exp\left(j\phi_{t_{N}}\right) \end{array}\right]$ and $\theta_{r}=\left[\begin{array}{c} \nu_{r_{1}}\exp\left(j\phi_{r_{1}}\right),\ldots ,\nu_{r_{N}}\exp\left(j\phi_{r_{N}}\right) \end{array}\right]$. Here, the amplitude coefficient and phase-shift for the transmission and reflection coefficient of the $n^{th}$ STAR-RIS element are denoted by $\nu_{t_{n}},\;\nu_{r_{n}}\in \left[0,1\right]$ and $\phi_{t_{n}},\;\phi_{r_{n}}\in \left[0,2\pi \right)$, $\forall n\in N=\{1,\ldots ,N_{s}\}$, respectively. Considering STAR-RIS is a lossless and passive device such that

(1) $${\left|\theta_{t_{n}}\right|}^{2}+{\left|\theta_{r_{n}}\right|}^{2}=1;{\left(\nu_{t_{n}}\right)}^{2}+{\left(\nu_{r_{n}}\right)}^{2}=1,\forall n\in N$$

To model the spatial correlation among antennas, the geometrical one-ring scattering model from [18] is adopted:

(2) $${\left[R_{k}\right]}_{i,j}=\frac{1}{2\Delta_{k}}\int_{\theta_{k}-\Delta_{k}}^{\theta_{k}+\Delta_{k}}e^{-j\frac{2*\pi }{\lambda }\Phi \left(\psi \right)\left(d_{i}-d_{j}\right)}\;d\psi$$

where $\Phi \left(\psi \right)=\left[cos\left(\psi \right),sin\left(\psi \right)\right],\;d_{i}={\left[x_{i},y_{i}\right]}^{T}$, $\theta_{k}$ is the azimuth angle of user *k* with respect to the orientation perpendicular to the array axis. $\Delta_{k}$ is the angular spread of departure to user *k*. The downlink channel of the direct BS-to-user *k* link is given as

(3) $$g_{k}^{BU}=U_{k}\Sigma_{k}^{\frac{1}{2}}z_{k}$$

where $\Sigma_{k}^{\frac{1}{2}}\in C^{r_{k}\times r_{k}}$ is the non-zero eigenvalues of the spatial correlation matrix, $U_{k}\in C^{M\times r_{k}}$ is the associated eigenvectors. We assume that all communication links are quasi-static block fading channels where $z_{k}\in C^{r_{k}\times 1}$ has independent and identical distributed $\mathrm{CN}\left(0,1\right)$ entries. The cascaded links $G_{k}^{BR_{s}U}$ and $g_{k}^{R_{s}U}$ can be modeled using the same approach.

Overall, the channel gain for the $k^{th}$ user is expressed as

(4) $$\begin{array}{c} g_{k}=\left\{\begin{array}{c} g_{k}^{BU}+\sum_{s\in S}G_{k}^{BR_{s}U}\theta_{s,r},k\in K_{r}\\ \sum_{s\in S}G_{k}^{BR_{s}U}\theta_{s,t},k\in K_{t} \end{array}\right. \end{array}$$

where $G_{k}^{BR_{s}U}=G^{BR_{s}}\mathrm{diag}\left(g_{k}^{R_{s}U}\right)$, $g_{k}^{BU}$ is the channel gain of direct BS-$k^{th}$ user link, $G^{BR_{s}}$ is the channel gain between the BS and the $s^{th}$ STAR-RIS, and $g_{k}^{R_{s}U}$ is the channel gain between the $s^{th}$ STAR-RIS and the $k^{th}$ user. The CSI for link is imperfectly known at BS, such that $G_{k}^{BR_{s}U}={\hat{G}}_{k}^{BR_{s}U}+\Delta G_{k}^{BR_{s}U},\;g_{k}^{BU}={\hat{g}}_{k}^{BU}+\Delta g_{k}^{BU}$, where ${\hat{G}}_{k}^{BR_{s}U},{\hat{g}}_{k}^{BU}$ represent the imperfect CSI and ${∥\Delta G_{k}^{BR_{s}U}∥}_{F}\leq \rho_{g,s,k},\;{∥\Delta g_{k}^{BU}∥}_{F}\leq \rho_{d,k}$ are the bounded channel estimation error.

In the considered STAR-RIS-aided C-HRSMA system, the BS splits the message at the $k^{th}$ user into the outer common part, inner common part and private part. The use of an outer common beamformer ensures that the dominant interference stream is uniformly managed across both the reflection and transmission spaces, thereby stabilizing the successive interference cancellation (SIC) process. Building upon this foundation, the employment of group-specific inner common streams enables the BS to fully leverage the distinct channel manipulation capabilities provided by the STAR-RIS for each respective space, which enhances the utilization of the system spatial degrees of freedom. Let $s_{c},s_{i_{k}}$, and $s_{p,k}$ represent the streams of outer common, inner common, and private parts of the message, respectively. $s=[s_{c},s_{t},s_{r},s_{1},\ldots ,s_{K}]$ represents the whole symbol streams transmitted at the BS, which are precoded by a precoding matrix $W=[w_{c},w_{t},w_{r},w_{1},\ldots ,w_{K}]$, which satisfies $E\left\{{\left|s_{k}\right|}^{2}\right\}=1$. Consequently, the signal $y_{k}$ received at the $k^{th}$ user can be expressed as

(5) $$\begin{array}{cc} y_{k}= & s_{c}g_{k}^{H}w_{c}+\sum_{l\in G}s_{l}g_{k}^{H}w_{l}+\sum_{j\in K}s_{j}g_{k}^{H}w_{j}\\ & +\sum_{j\in A}\sum_{s\in S}c_{s}\theta_{s,i_{k}}^{H}G_{k}^{BR_{s}U^{H}}w_{j}+n_{k} \end{array}$$

where $i_{k}\in G=\left\{T,R\right\}$ represents the group to which user *k* belongs to, A=K∪G∪{c} and $n_{k}∼\mathrm{CN}\left(0,\sigma_{k}^{2}\right)$ is the additive white Gaussian noise (AWGN).

When decoding the outer common stream, both the inner common stream and the private streams are treated as interference. Subsequently, the outer common stream is canceled using SIC technology, and the inner common stream is then decoded by treating the private streams as interference. Finally, the private streams are decoded. Therefore, the SINR of the common message and private message for user *k* can be written as follows:

(6) $$\begin{array}{cc} \; & \gamma_{c,k}=\frac{{\left|g_{k}^{H}w_{c}\right|}^{2}}{I_{k}},\gamma_{ic,k}=\frac{{\left|g_{k}^{H}w_{i_{k}}\right|}^{2}}{I_{k}-{\left|g_{k}^{H}w_{i_{k}}\right|}^{2}}\\ \; & \gamma_{p,k}=\frac{{\left|g_{k}^{H}w_{k}\right|}^{2}}{\sum_{j\in K,j\neq k}{\left|g_{k}^{H}w_{j}\right|}^{2}+\sigma_{k}^{2}} \end{array}$$

where $I_{k}=\sum_{l\in G}{\left|g_{k}^{H}w_{l}\right|}^{2}+\sum_{j\in K}{\left|g_{k}^{H}w_{j}\right|}^{2}+\sigma_{k}^{2}$.

The achievable rates for the corresponding outer common, inner common, and private streams, respectively, at the $k^{th}$ user over bandwidth *B* are provided by

(7) $$\begin{array}{cc} & R_{k}^{c}=B\log_{2}\left(1+\gamma_{c,k}\right),R_{k}^{i_{k}}=B\log_{2}\left(1+\gamma_{ic,k}\right)\\ & R_{k}^{p}=B\log_{2}\left(1+\gamma_{p,k}\right) \end{array}$$

To ensure that all users can decode the common data correctly, we have $R_{0}^{c}=\min\left\{R_{1}^{c},R_{2}^{c},\ldots ,R_{K}^{c}\right\},\;\sum_{k\in K}R_{c,k}\leq R_{0}^{c},\;R_{c,k}\geq 0,$ $R_{0}^{l}=\min\left\{R_{1}^{l},R_{2}^{l},\ldots ,R_{K_{l}}^{l}\right\},$ $\sum_{k\in K_{l}}R_{l,k}\leq R_{0}^{l},\;R_{l,k}\geq 0,$ l∈{T,R}. After decoding the private stream and remove it from the received signal using SIC, the achievable transmission rate for decoding *c* is

(8) $$R_{k}^{sr}=\sum_{s\in S}\frac{B}{L}\log_{2}\left(1+\frac{L}{\sigma_{k}^{2}}\left(|\theta_{s,i_{k}}^{H}G_{k}^{BR_{s}U^{H}}\sum_{k\in A}w_{k}{|}^{2}\right)\right)$$

where *L* is the ratio between the primary transmission symbol periods and the secondary transmission symbol periods.

Thus, the total rate of user *k* is

(9) $$R_{k}=R_{c,k}+R_{i_{k},k}+R_{k}^{p}+R_{k}^{sr}$$

## 3. Problem Formulation

A weighted sum of objectives is commonly adopted to accomplish tradeoffs between competing goals. To this end, the multi-objective optimization problem is formulated as [19]

(10a) $$\begin{array}{cc} (P0): & \min_{W,\theta_{s,t},\theta_{s,r}}\tau \frac{\delta_{EE}P^{total}}{\sum_{k\in K}R_{k}}+\left(1-\tau \right)\frac{\delta_{SE}B}{\sum_{k\in K}R_{k}} \end{array}$$

(10b) $$\begin{array}{cc} \; & {∥\Delta G_{k}^{BR_{s}U}∥}_{F}\leq \rho_{g,s,k},{∥\Delta g_{k}^{BU}∥}_{F}\leq \rho_{d,k} \end{array}$$

(10c) $$\begin{array}{cc} \; & \sum_{k\in K}w_{k}^{H}w_{k}+\sum_{l\in G}w_{l}^{H}w_{l}+w_{c}^{H}w_{c}\leq P^{max} \end{array}$$

(10d) $$\begin{array}{cc} \; & \sum_{k\in K_{i_{k}}}R_{i_{k},k}\leq R_{0}^{i_{k}},\sum_{k\in K}R_{c,k}\leq R_{0}^{c} \end{array}$$

(10e) $$\begin{array}{cc} \; & R_{c,k}+R_{i_{k},k}+R_{k}^{p}+R_{k}^{sr}\geq r_{k}^{min},\forall k\in K \end{array}$$

(10f) $$\begin{array}{cc} \; & R_{k}^{p},R_{ic,k},R_{k}\geq 0,\forall k\in K \end{array}$$

(10g) $$\begin{array}{cc} \; & {\left|\theta_{s,t_{n}}\right|}^{2}+{\left|\theta_{s,r_{n}}\right|}^{2}=1,\forall s\in S,\forall n\in N \end{array}$$

where τ is employed as tradeoff parameter to represent the tradeoff in this multi-objective optimization problem, and $\delta_{EE}$ and $\delta_{SE}$ are normalization factors. The constraint (10c) represents the maximum transmit power at the BS ensuring the overall transmit power does not exceed the total available power $P^{max}$. The constraint (10d) indicates the common rate criterion. With $r_{k}^{min}$ standing for the minimal data rate of $k^{th}$ user, constraint (10e) guarantees the QoS data rate. The constraint (10g) confines the amplitude coefficient and phase coefficient of each STAR-RIS.

In order to deal with the robust joint beamforming design problem in (10a), we transform the constraints into more simplified form using S-procedure and linear matrix inequalities (LMIs). The fractional objective function (10a) can be transformed into the following equivalent convex function using the following lemma.

**Lemma** **1** (Dinkelbach’s approach [20])**.** *Let $x^{*}$ be a solution of the following problem:*

$$\min_{x}\frac{f(x)}{g(x)}\;s.t.\;x\in X.$$

*where f(x) and g(x) are the convex and concave functions with respect to x, respectively, while X denotes the feasible region of x. Then, the minimum objective function value $\omega^{*}=f\left(x^{*}\right)/g\left(x^{*}\right)$ can be obtained if and only if*

$$\min_{x}\left\{f\left(x\right)-\omega^{*}g\left(x\right)\;|\;x\in X\right\}=f\left(x^{*}\right)-\omega^{*}g\left(x^{*}\right)=0.$$

By applying Lemma 1 to (10a), with the numerator identified as $f\left(x\right)=\tau \delta_{EE}P^{total}+\left(1-\tau \right)\delta_{SE}B$ and the denominator as $g\left(x\right)=\sum_{k\in K}R_{k}$, problem (10a) can be reformulated as [21]

(11) $$F_{EE-SE}=\tau \delta_{EE}P^{total}+\left(1-\tau \right)\delta_{SE}B-\omega \sum_{k\in K}R_{k}$$

where ω is the parameter of the iterative Dinkelbach algorithm.

We rewrite the problem in (10a) using auxiliary variables $\alpha =\left\{\alpha_{c,k},\alpha_{i_{k},k},\alpha_{k},\forall k\right\},\beta =\left\{\beta_{c,k},\beta_{i_{k},k},\beta_{k},\forall k\right\}$ as [22]

(12a) $$\begin{array}{cc} (P1): & \min_{\begin{array}{c} W,\theta_{s,t},\theta_{s,r},\\ \alpha ,\beta ,R_{\mathrm{ic}},R_{c} \end{array}}F_{EE-SE}\left(\alpha \right)\\ s.t. & \left(10b),(10c),(10d),(10e),(10f),(10g\right) \end{array}$$

(12b) $$\begin{array}{cc} \; & {\left|g_{k}^{H}w_{k}\right|}^{2}/\beta_{k}\geq \alpha_{k} \end{array}$$

(12c) $$\begin{array}{cc} \; & \sum_{j\in K,j\neq k}{\left|g_{k}^{H}w_{j}\right|}^{2}+\sigma_{k}^{2}\leq \beta_{k} \end{array}$$

(12d) $$\begin{array}{cc} \; & {\left|g_{k}^{H}w_{i_{k}}\right|}^{2}/\beta_{i_{k},k}\geq \alpha_{i_{k},k} \end{array}$$

(12e) $$\begin{array}{cc} \; & IN_{k}-{\left|g_{k}^{H}w_{i_{k}}\right|}^{2}\leq \beta_{i_{k},k} \end{array}$$

(12f) $$\begin{array}{cc} \; & {\left|g_{k}^{H}w_{c}\right|}^{2}/\beta_{k}\geq \alpha_{c} \end{array}$$

(12g) $$\begin{array}{cc} \; & IN_{k}\leq \beta_{c} \end{array}$$

(12h) $$\begin{array}{cc} \; & |\theta_{s,i_{k}}^{H}G_{k}^{BR_{s}U^{H}}\sum_{k\in A}w_{k}{|}^{2}\geq \frac{\sigma_{k}^{2}}{L}\alpha_{sr,k},\forall k\in K \end{array}$$

Under the channel estimation error constraint (10b), the expressions in (12b), (12d), and (12f) can be linearly approximated using their lower bound such for any $t^{th}$ iteration of SCA and transformed into quadratic constraints.

(13) hkHPkhk+2Re{qkHhk}+rk≥αk

(14) hk=ΔgkBUH,…,vecHΔGkBRSUHPk=2Re{P˜k}βkt−βkP¯kP¯kHβkt2qk=q˜kβkt−βkq¯kβkt2rk=2Re{g^ktHwktwkHg^k}βkt−βkg^ktHwktwktHg^ktβkt2

The specific expressions for each component of the variables $P_{k}$, $q_{k}$, and $r_{k}$ are given as follows:

(15) $$\begin{array}{cc} \; & {\tilde{P}}_{k}=\left[\begin{array}{c} w_{k}^{\left(t\right)}\\ \theta_{1,i_{k}}^{{\left(t\right)}^{*}}⊗w_{k}^{\left(t\right)}\\ \vdots \\ \theta_{S,i_{k}}^{{\left(t\right)}^{*}}⊗w_{k}^{\left(t\right)} \end{array}\right]\left[w_{k}^{H},\theta_{1,i_{k}}^{T}⊗w_{k}^{H},\ldots ,\theta_{S,i_{k}}^{T}⊗w_{k}^{H}\right]\\ \; & {\bar{P}}_{k}={\left[w_{k}^{\left(t\right)},\theta_{1,i_{k}}^{{\left(t\right)}^{*}}⊗w_{k}^{\left(t\right)},\ldots ,\theta_{S,i_{k}}^{{\left(t\right)}^{*}}⊗w_{k}^{\left(t\right)}\right]}^{H}\\ \; & {\tilde{q}}_{k}=\left[\begin{array}{c} w_{k}^{\left(t\right)}w_{k}^{H}{\hat{g}}_{k}\\ vec\left(w_{k}^{\left(t\right)}w_{k}^{H}{\hat{g}}_{k}\theta_{1,i_{k}}^{{\left(t\right)}^{H}}\right)\\ \vdots \\ vec\left(w_{k}^{\left(t\right)}w_{k}^{H}{\hat{g}}_{k}\theta_{S,i_{k}}^{{\left(t\right)}^{H}}\right) \end{array}\right]+\left[\begin{array}{c} w_{k}w_{k}^{{\left(t\right)}^{H}}{\hat{g}}_{k}^{\left(t\right)}\\ vec\left(w_{k}w_{k}^{{\left(t\right)}^{H}}{\hat{g}}_{k}^{\left(t\right)}\theta_{1,i_{k}}^{{\left(t\right)}^{H}}\right)\\ \vdots \\ vec\left(w_{k}w_{k}^{{\left(t\right)}^{H}}{\hat{g}}_{k}^{\left(t\right)}\theta_{S,i_{k}}^{{\left(t\right)}^{H}}\right) \end{array}\right]\\ \; & {\bar{q}}_{k}=\left[\begin{array}{c} w_{k}^{\left(t\right)}w_{k}^{{\left(t\right)}^{H}}{\hat{g}}_{k}^{\left(t\right)}\\ vec\left(w_{k}^{\left(t\right)}w_{k}^{{\left(t\right)}^{H}}{\hat{g}}_{k}^{\left(t\right)}\theta_{1,i_{k}}^{{\left(t\right)}^{H}}\right)\\ \vdots \\ vec\left(w_{k}^{\left(t\right)}w_{k}^{{\left(t\right)}^{H}}{\hat{g}}_{k}^{\left(t\right)}\theta_{S,i_{k}}^{{\left(t\right)}^{H}}\right) \end{array}\right] \end{array}$$

where ${\hat{g}}_{k}={\hat{g}}_{k}^{BU}+\sum_{s\in S}{\hat{G}}_{k}^{BR_{s}U}\theta_{s,i_{k}}$, ${\hat{g}}_{k}^{\left(t\right)}={\hat{g}}_{k}^{BU}+\sum_{s\in S}{\hat{G}}_{k}^{BR_{s}U}\theta_{s,i_{k}}^{\left(t\right)}$. By using (10b) and the general S-procedure, the quadratic inequality in (13) can be transformed into LMI as

(16) $$\left[\begin{array}{cc} Q_{k,\alpha }+P_{k} & q_{k}\\ q_{k}^{H} & {\bar{r}}_{k} \end{array}\right]⪰0$$

where $\upsilon_{k}$ is the positive auxiliary variable, $Q_{k,\alpha }=\mathrm{diag}\left(\upsilon_{k,d}1_{M},\ldots ,\upsilon_{k,S}1_{MN}\right),$ and ${\bar{r}}_{k}=r_{k}-\alpha_{k}-\upsilon_{k,d}\rho_{d,k}^{2}-\sum_{s\in S}\upsilon_{k,s}\rho_{g,s,k}^{2}$. Let $1_{M}$ denote the row vector of size 1×M whose elements are all one.

Furthermore, the inequalities (12c), (12e), (12g) can be rewritten as

(17) $$\left[\begin{array}{ccc} {\bar{\beta }}_{k} & {\hat{g}}_{k}^{H}{\tilde{w}}_{k} & 0^{T}\\ {\tilde{w}}_{k}^{H}{\hat{g}}_{k} & I & {\hat{w}}_{k}\\ 0 & {\hat{w}}_{k}^{H} & Q_{k,\beta } \end{array}\right]⪰0$$

where ${\tilde{w}}_{k}=\left\{w_{k},k\in K/k\right\},\;{\tilde{w}}_{c}=\left\{w_{l},l\in K\cup G\right\},\;\;{\tilde{w}}_{i_{k}}=\left\{w_{l},l\in K\cup G\setminus l\right\},$ ${\bar{\beta }}_{k}=\beta_{k}-\sigma_{k}^{2}-\epsilon_{k,d}-\sum_{s\in S}\epsilon_{k,s}N,$ ${\hat{w}}_{k}=\left[\rho_{d,k}{\tilde{w}}_{k}^{H},\ldots ,\rho_{g,S,k}{\tilde{w}}_{k}^{H}\right],$ $k^{\prime }\in \left\{K/k\right\},$ $Q_{k,\beta }=\mathrm{diag}\left(\epsilon_{k,d}1_{M-1},\ldots ,\epsilon_{k,S}1_{M-1}\right)$ and ϵ is the auxiliary variable.

Similarly, constraint (12h) can be transformed into the following form:

(18) $$\left[\begin{array}{cc} Q_{sr,k}+P_{sr,k} & q_{sr,k}\\ q_{sr,k}^{H} & {\bar{r}}_{sr,k}-\frac{\sigma_{k}^{2}}{L}\alpha_{sr,k} \end{array}\right]⪰0$$

where $w_{sr}=\sum_{k\in A}w_{k},\;{\bar{r}}_{sr,k}=r_{sr,k}-\upsilon_{k,d}\rho_{d,k}^{2}-\sum_{s\in S}\upsilon_{k,s}\rho_{g,s,k}^{2}$.

Ultimately, the problem $\left(P1\right)$ can be approximately rewritten at the $a^{th}$ SCA iteration using (16)–(18) as

(19a) $$\begin{array}{cc} (P2): & \min_{\begin{array}{c} W,\theta ,\alpha ,\\ \beta ,R_{\mathrm{ic}},R_{c} \end{array}}F_{EE-SE}\left(\alpha \right)\\ s.t. & \left(10c),(10d),(10e),(10f),(10g\right)\\ & \left(16),(17),(18\right) \end{array}$$

(19b) $$\begin{array}{cc} \; & \upsilon \geq 0,\epsilon \geq 0 \end{array}$$

To address the coupling of precoder at the BS and the passive beamforming at the STAR-RIS, the AO framework is utilized to efficiently decouple and solve the optimization problem.

### 3.1. Precoder Design

The problem $\left(P2\right)$ can be reformulated as follows, given reflection and transmission matrices at the STAR-RIS.

(20a) $$\begin{array}{cc} \; & \min_{\begin{array}{c} W,\alpha ,\beta \\ R_{\mathrm{ic}},R_{c} \end{array}}F_{EE-SE}\left(\alpha \right)\\ s.t. & \left(10c),(10d),(10e),(10f\right)\\ & \left(16),(17),(18\right) \end{array}$$

(20b) $$\begin{array}{cc} \; & \upsilon \geq 0,\epsilon \geq 0\\ \; & \theta_{t}=\theta_{t}^{\left(t\right)},\theta_{r}=\theta_{r}^{\left(t\right)} \end{array}$$

Hence, (20a) is a convex optimization problem with semi-definite programming (SDP) and affine constraints, which can be solved using the CVX toolbox.

### 3.2. Passive Beamforming Design

Finally, the unity-modulus convex constraint () renders the problem P2 non-convex. Consequently, we relax the unity-modulus constraints as ${\left|\theta_{t_{n}}\right|}^{2}+{\left|\theta_{r_{n}}\right|}^{2}\leq 1$, and then employ the penalty method to reformulate the problem. However, the newly added term in the objective function is non-convex. This non-convexity can be addressed by approximating the term using the first-order Taylor expansion as Hθtn,θrn=2Reθtntθtn∗+2Reθrntθrn∗−θtnt2−θrnt2−1. The passive beamforming problem can be rewritten as

(21a) $$\begin{array}{cc} \; & \min_{\begin{array}{c} \theta ,\alpha \end{array}}F_{EE-SE}\left(\alpha \right)+p\sum_{n\in N}\left(H\left(\theta_{t_{n}},\theta_{r_{n}}\right)\right)\\ s.t. & \left(10c),(10d),(10e),(10f\right)\\ & \left(16),(17),(18\right) \end{array}$$

(21b) $$\begin{array}{cc} \; & \upsilon \geq 0,\epsilon \geq 0 \end{array}$$

(21c) $$\begin{array}{cc} \; & {\left|\theta_{t_{n}}\right|}^{2}+{\left|\theta_{r_{n}}\right|}^{2}\leq 1,\forall n\in N\\ \; & w=w^{\left(t\right)} \end{array}$$

where *p* is the penalization factor. Problem (21a) is a semi-definite regularization problem for which stationary points can be obtained using the CVX toolbox.

Ultimately, Algorithm 1 summarizes the complete algorithm flow for solving the problem $\left(P0\right)$.
**Algorithm 1** AO-Based Joint Beamforming Design1:**Input**: $w^{\left(0\right)},\;\theta_{t}^{\left(0\right)},\;\theta_{r}^{\left(0\right)},\;\delta ,\;\zeta_{dim},\;T_{max},\;A_{max}$2:**Initialization**: $a=0,\;\omega^{\left(0\right)}=0.25$3:**while** $a\leq A_{max}$ **and** $|F_{EE-SE}^{\left(a\right)}|\geq \;\zeta_{dim}$:4:     **while** $t\leq T_{max}$ **and** $\left|F_{EE-SE}^{\left(t-1\right)}-F_{EE-SE}^{\left(t\right)}\right|\geq \delta$:5:        Solve (20a) for active precoder design6:        Solve (21a) for passive beamforming design7:        t←t+18:     **end while**9:     $\omega^{\left(a+1\right)}\leftarrow \tau \frac{\delta_{EE}P^{\left(a+1\right)}}{\sum_{k\in K}R_{k}^{\left(a+1\right)}}+\left(1-\tau \right)\frac{\delta_{SE}B}{\sum_{k\in K}R_{k}^{\left(a+1\right)}}$10:     a←a+111:**end while**12:**output**: $W,\theta ,\omega^{\left(a+1\right)}$

### 3.3. Computational Cost of Algorithm

In the proposed joint beamforming design scheme based on an AO framework, the results of the active beamforming algorithm are sequentially utilized as inputs for the passive beamforming algorithm. The worst-case computational complexity of a general SDP problem is given as follows [22]:

$$O\left(\left(v^{3}+v^{2}\sum_{c=1}^{C}l_{c}^{2}+\sum_{c=1}^{C}l_{c}^{3}\right)\sqrt{\sum_{c=1}^{C}l_{c}}\right)$$

where *C* is the number of SDP constraints of size $l_{c}$, and *v* is the number of variables. In the active precoder design subproblem, $l_{1}=MNS+M+1$, $l_{2}=MS+K+2$, $l_{3}=MN_{s}S+M+1$, and v=KM+4(K+3), where *M* is the number of BS antennas, $N_{s}$ is the number of RIS elements, *S* is the number of RIS, and *K* is the number of users. Thus, after ignoring constant factors and lower-order terms, the computational complexity of the active precoder design is approximated as $O\left(\left({\left(KM+4K\right)}^{3}+2M^{2}N_{s}^{2}S^{2}{\left(KM+4K\right)}^{2}+2M^{3}N_{s}^{3}S^{3}\right)\sqrt{2MN_{s}S+MS}\right)$. Similarly, in the passive beamforming subproblem, $l_{1}=MN_{s}S+M+1$, $l_{2}=N_{s}S+K+2$, $l_{3}=MN_{s}S+M+1$, and $v=N_{s}+4\left(K+3\right)$. The worst-case time complexity for passive beamforming is approximated as $O\left(\left({\left(N_{s}+4K\right)}^{3}+2M^{2}N_{s}^{2}S^{2}{\left(N_{s}+4K\right)}^{2}+2M^{3}N_{s}^{3}S^{3}\right)\right.$$\left.\sqrt{2MN_{s}S+N_{s}S}\right)$. The joint solution for the precoder and phase-shifter design converges in $T^{max}$ iterations at most. Therefore, the worst-case computational complexity of the AO framework can be approximated as follows:

(22) $$\begin{array}{cc} O(T^{max}( & \sqrt{2MN_{s}S+MS}\left(2M^{2}N_{s}^{2}S^{2}{\left(KM+4K\right)}^{2}+2M^{3}N_{s}^{3}S^{3}\right)\\ & +\sqrt{2MN_{s}S+N_{s}S}\left(2M^{2}N_{s}^{2}S^{2}{\left(N_{s}+4K\right)}^{2}+2M^{3}N_{s}^{3}S^{3}\right))) \end{array}$$

Given that the proposed iterative optimization framework, which integrates Dinkelbach’s method, precoder optimization, and phase-shifter design, converges within a maximum of $A_{max}T_{max}$ iterations facilitated by the SCA technique, the worst-case computational complexity of the Algorithm 1 can be consequently approximated as follows:

(23) $$\begin{array}{cc} O(A^{max}T^{max}( & \sqrt{2MN_{s}S+MS}\left(2M^{2}N_{s}^{2}S^{2}{\left(KM+4K\right)}^{2}+2M^{3}N_{s}^{3}S^{3}\right)\\ & +\sqrt{2MN_{s}S+N_{s}S}\left(2M^{2}N_{s}^{2}S^{2}{\left(N_{s}+4K\right)}^{2}+2M^{3}N_{s}^{3}S^{3}\right))) \end{array}$$

In [22], the benchmark robust beamforming algorithm for the STAR-RIS-aided NOMA system exhibits a computational complexity of $O(A^{\max}(\left({\left(MK+4K\right)}^{3}+{\left(MN+M+K\right)}^{3}\right)\sqrt{MN+M+K}$ + $\left(\left(64{\left(N+K\right)}^{3}+{\left(MN+N+K\right)}^{3}\right)\sqrt{MN+N+K})\right))$. Our proposed C-HRSMA algorithm achieves superior system performance while maintaining a comparable level of complexity.

## 4. Results

This section demonstrates the performance of the STAR-RIS-aided C-HRSMA system through detailed computer simulations. Moreover, the comparison algorithms considered in the simulations involve the STAR-RIS-aided NOMA system [22] and the algorithm of RIS-aided RSMA SR system [1].

The parameters of the channel model are set in accordance with [18]. The other parameters are as follows: $B=100\;\mathrm{MHz},\;K_{t}=K_{r}=2,\;M=4,\;N=10,\;S=2,\;\sigma_{k}^{2}=-50\;\mathrm{dBm},$ $R_{k}^{min}=0.5\;\mathrm{bit}/s,$ $P^{max}=40\;\mathrm{dBm},\;L=10$, and $\rho_{g}=\rho_{d}=0.01$.

**Figure 2:**
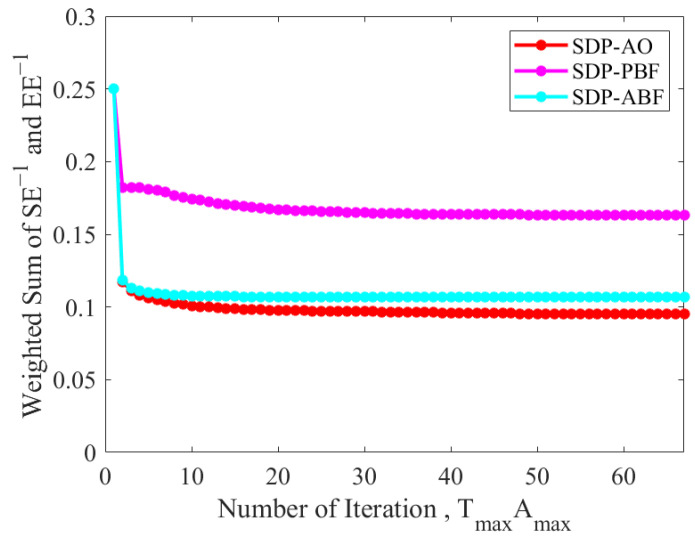
Convergence analysis of the proposed algorithm.

As illustrated in Figure 2, the individual convergence of the active beamforming at the BS, the passive beamforming at the STAR-RIS, and the AO process was analyzed. The results indicate that the passive beamforming converges within 37–39 iterations (requiring 6.84 s), the active beamforming reaches convergence within 27–29 iterations (requiring 5.18 s), and the overall algorithm attains convergence to a stable point within 57–60 iterations, with a total runtime of 10.23 s.

**Figure 3:**
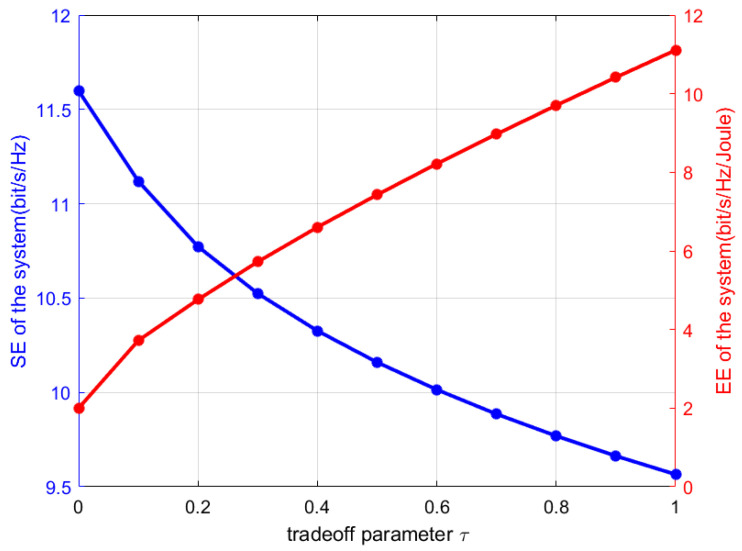
Impact of tradeoff parameter.

Figure 3 illustrates that the tradeoff between SE and EE can be balanced by adjusting tradeoff parameter τ. With an increasing tradeoff parameter τ, the objective function prioritizes EE enhancement, leading to higher EE but lower SE in the system. When τ=0, the optimization problem is equivalent to an exclusive SE maximization problem. When τ=1, the optimization problem is equivalent to an exclusive EE maximization problem.

**Figure 4:**
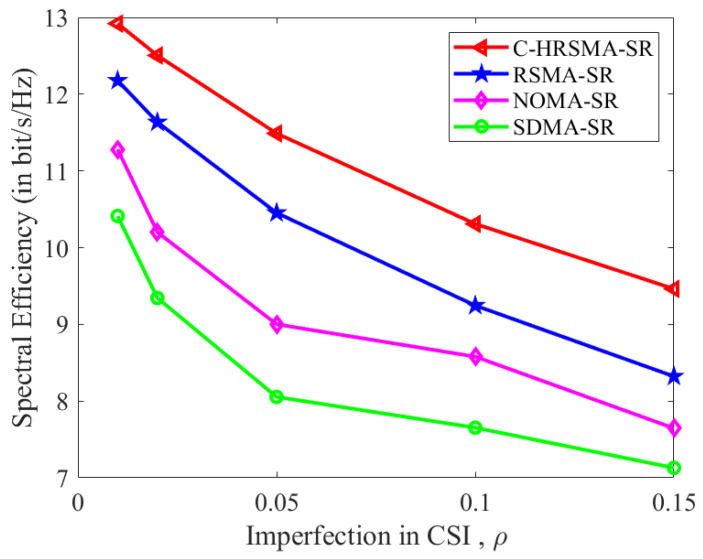
SE versus Imperfection in CSI.

**Figure 5:**
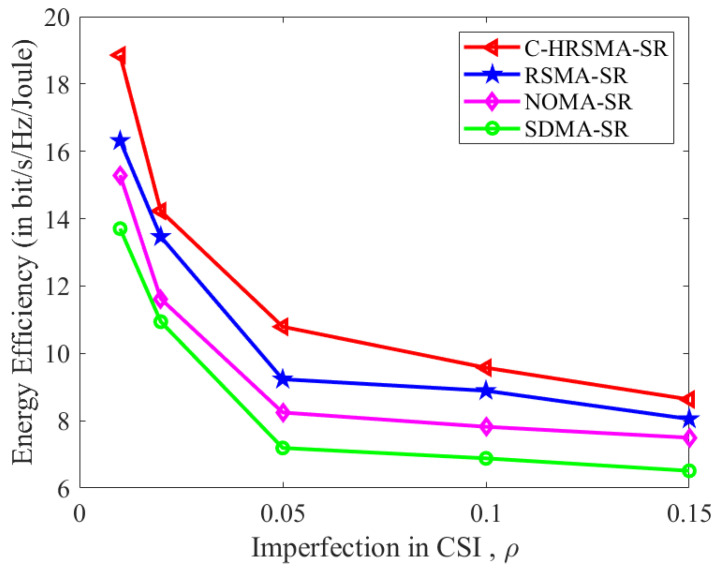
EE versus Imperfection in CSI.

**Figure 6:**
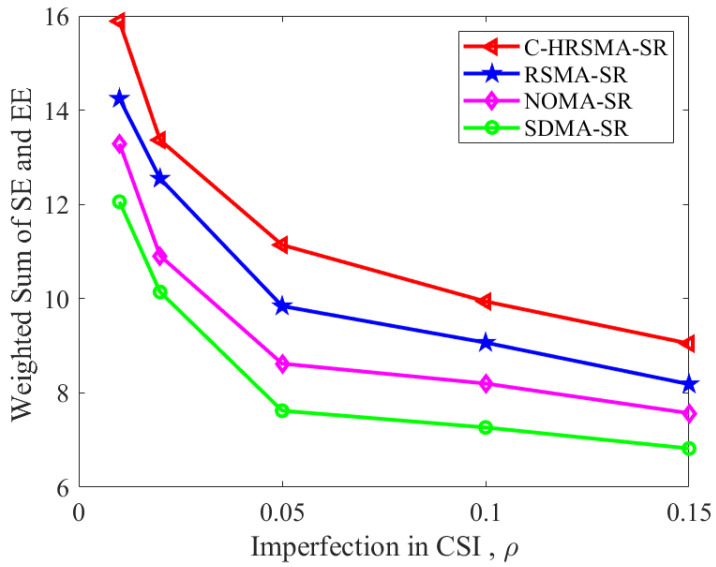
Weighted sum versus Imperfection in CSI.

Figure 4, Figure 5 and Figure 6 depict the impact of channel estimation errors. As shown in Table 2, the proposed C-HRSMA system achieves improvements of 10.03% in SE and 16.33% in EE, respectively, compared with the RSMA system. The increase in ICSI deteriorates the performance of SR systems. Nevertheless, among all SR systems, C-HRSMA exhibits greater robustness and is less susceptible to the adverse effects of ICSI. C-HRSMA exhibits superior robustness against ICSI compared with space-division multiple access (SDMA). Notably, the system performance degradation of the robustly optimized C-HRSMA is less pronounced than that of SDMA as the channel estimation error variance increases.

Figure 6 demonstrates that the weighted sum consistently reflects C-HRSMA’s superior advantage in both SE and EE as the ICSI increases.

**Figure 7:**
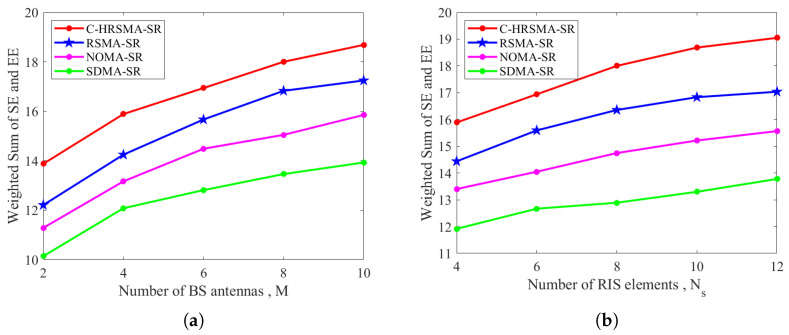
Weighted sum versus antenna scale: (**a**) Weighted sum versus number of BS antennas. (**b**) Weighted sum versus number of STAR-RIS elements.

Figure 7 illustrates that the proposed C-HRSMA scheme surpasses the other comparison schemes as the number of BS antennas and RIS elements increases. In particular, with the increase in BS antennas, all schemes exhibit enhanced performance, attributed to more efficient beamforming design. Furthermore, an increase in the number of STAR-RIS elements enables the STAR-RIS to possess superior wireless channel manipulation capabilities. Consequently, the performance improvement of the proposed C-HRSMA scheme is more pronounced as the number of STAR-RIS elements increases.

## 5. Conclusions

This paper investigates the robust tradeoff between SE and EE within a C-HRSMA system. The objective is to optimize the weighted sum of SE and EE through the joint optimization of the users’ common rate allocation, the BS transmit beamforming vector, and the RIS passive beamforming matrix. Initially, the optimization problem is reformulated using Dinkelbach’s approach to convert the fractional objective into an equivalent subtractive form, simultaneously minimizing power consumption and maximizing achievable rate. Subsequently, an AO framework is employed to decouple the coupled variables, where SCA is iteratively applied to address the non-convex subproblems. Finally, numerical simulations verify the proposed method’s superior performance in terms of SE and EE and robustness compared with benchmark SR systems under ICSI, demonstrating the capability to shift optimization emphasis by modifying the tradeoff parameter.

## Figures and Tables

**Table 1:** List of symbols.

Symbols	Description	Symbols	Description
*M*	Number of BS antennas	*K*	Total number of users
*S*	Number of STAR-RISs	S	Set of STAR-RISs
$N_{s}$	Number of elements per STAR-RIS	N	Set of STAR-RIS elements
$K_{t}$	Number of T-users	$K_{t}$	Set of T-users
$K_{r}$	Number of R-users	$K_{r}$	Set of R-users
$\Theta_{t}$	Transmission coefficient matrix	$\Theta_{r}$	Reflection coefficient matrix
$\theta_{t}$	Transmission coefficient vector	$\theta_{r}$	Reflection coefficient vector
$v_{t_{n}}$	Transmit amplitude coefficient	$v_{r_{n}}$	Reflect amplitude coefficient
$\phi_{t_{n}}$	Transmit phase-shift	$\phi_{r_{n}}$	Reflect phase-shift
$g_{k}^{BU}$	BS-to-user *k* channel	$G_{k}^{BR_{s}U}$	BS-RIS *s*-user *k* channel
${\hat{g}}_{k}^{BU}$	Estimated BS-to-user *k* channel	${\hat{G}}_{k}^{BR_{s}U}$	Estimated cascaded channel
$\rho_{d,k}$	Bounded error of $g_{k}^{BU}$	$\rho_{g,s,k}$	Bounded error of $G_{k}^{BR_{s}U}$
$s_{c}$	Outer common stream	*B*	System bandwidth
$s_{i_{k}}$	Inner common stream	$s_{p,k}$	Private stream (for user *k*)
W	Precoding matrix	$w_{c}$	Outer common precoder
$w_{k}$	Private precoder for user *k*	$P^{max}$	Maximum BS transmit power
$y_{k}$	Received signal at user *k*	$\sigma_{k}^{2}$	Noise power at $k^{th}$ user
$\gamma_{c,k}$	SINR for outer common stream	$R_{k}$	Total rate of user *k*
$\gamma_{ic,k}$	SINR for inner common stream	$\gamma_{p,k}$	SINR for private stream
τ	EE-SE tradeoff parameter	$r_{k}^{min}$	Minimum QoS rate for user *k*

**Table 2:** Performance comparison and quantified gains at ρ=0.05.

Scheme	SE (bit/s/Hz)	EE (bit/s/Hz/Joule)	SE Gain (over RSMA-SR)	EE Gain (over RSMA-SR)
**C-HRSMA-SR**	**11.52**	**10.83**	**10.03%**	**16.33%**
RSMA-SR [1]	10.47	9.31	—	—
NOMA-SR [22]	9.03	8.37	—	—
SDMA-SR	8.13	7.26	—	—

## Data Availability

Data are contained within the article.
